# Exosomal non-coding RNAs: orchestrators of intercellular crosstalk in the prostate cancer tumor microenvironment

**DOI:** 10.3389/fimmu.2025.1644861

**Published:** 2025-11-21

**Authors:** Huanglin Duan, Baisheng Xu, Peiyue Luo, Tao Chen, Jun Zou

**Affiliations:** 1 Department of Urology, The First People's Hospital of Xiushui, Jiujiang, Jiangxi, China; 2 The First Clinical College, Gannan Medical University, Ganzhou, Jiangxi, China; 3 Department of Otorhinolaryngology, The Affiliated Fengcheng Hospital of Yichun University, Fengcheng, Jiangxi, China

**Keywords:** exosomes, non-coding RNA, prostate cancer, tumor microenvironment, immune microenvironment

## Abstract

Prostate cancer (PCa) remains a leading cause of cancer-related mortality in men worldwide, primarily due to its propensity for therapy resistance and metastasis. Emerging evidence underscores that exosomes, nanoscale extracellular vesicles, act as critical mediators of intercellular communication within the tumor microenvironment (TME), particularly via the non-coding RNAs (ncRNAs) they transport. These molecules include microRNAs (miRNAs), circular RNAs (circRNAs), and long non-coding RNAs (lncRNAs). Exosomal ncRNAs drive tumor progression, immune evasion, and therapy resistance by reprogramming neighboring stromal cells, immune cells, and malignant cells. This review systematically examines the multifaceted roles of exosomal ncRNAs in remodeling the prostate cancer tumor microenvironment, focusing on their communication between tumor cells, tumor-stromal cells (including immune cells), and within the pre-metastatic niche preceding bone metastasis. We emphasize their mechanisms of action and clinical relevance. These findings position exosomal ncRNAs as central drivers of prostate cancer progression, revealing novel diagnostic and therapeutic opportunities. Future research must address challenges in standardizing exosome isolation techniques, resolving spatiotemporal heterogeneity, and advancing clinical translation to fully realize the potential of exosome-based strategies in precision oncology.

## 1 Introduction

Prostate cancer (PCa) is the second most prevalent malignant tumor in males globally, primarily affecting individuals aged 45–60, as evidenced by recent epidemiological data (1). As reported by the International Agency for Research on Cancer’s GLOBOCAN database, PCa accounted for over 1.4 million new cases and 375,000 deaths worldwide in 2020 and demonstrates a rising trend (1–3). Notably, managing advanced metastatic and castration-resistant prostate cancer (CRPC) remains a critical clinical challenge (4). While early-stage localized PCa is curable via surgery or radiotherapy (5-year survival rate approaching 100%) (5), recent research evaluating robot-assisted laparoscopic radical prostatectomy reported biochemical recurrence in approximately 10.19% of T3aN0-stage patients within 15.22 months post-surgery (6). However, the 5-year survival rate plummets to 30% once distant metastasis occurs (5). Despite advancements in surgical, radiotherapeutic, and endocrine therapies for PCa, substantial challenges persist. Tumor heterogeneity leads to marked variability in therapeutic responses, particularly to chemotherapy and endocrine interventions (7). Prolonged treatment durations exacerbate therapy-related side effects and complications, adversely affecting patients’ psychological and physiological well-being, treatment adherence, and quality of life (8). These challenges underscore the necessity for deeper mechanistic investigations into PCa pathogenesis to refine therapeutic strategies.

Cancer is widely recognized as a dynamic evolutionary process driven by intricate interactions between tumor cells and the tumor microenvironment (TME) (9, 10). Emerging studies have established that prostate cancer invasiveness is closely linked to its distinctly heterogeneous TME (11, 12). The TME comprises tumor cells, cancer-associated fibroblasts (CAFs), immune cells, vascular endothelial cells, and extracellular matrix (ECM). Non-cellular components, including growth factors, extracellular vesicles, and acellular ECM, collectively form a dynamic, pro-metastatic, and therapy-resistant ecosystem (13, 14). Exosomes, a subtype of extracellular vesicles, have emerged as pivotal mediators of intercellular communication within the TME, owing to their ability to regulate tumor progression via transport of non-coding RNAs (ncRNAs) (15, 16). These nanoscale vesicles, which are actively secreted by cells and range from 30 to 150 nm in diameter, are not merely cellular waste products but originate from multivesicular bodies (MVBs). The endosomal membrane buds inward to form an MVB, which contains numerous intraluminal vesicles; this MVB subsequently fuses with the plasma membrane, releasing these vesicles into the extracellular space as exosomes (17). Consequently, exosomes carry a diverse molecular “cargo” derived from their parent cells, including specific membrane proteins (e.g., CD9, CD63, CD81), cytoplasmic proteins, and nucleic acids (e.g., ncRNAs). Among these components, surface integrins and tissue-specific markers (e.g., PSMA) facilitate the targeted delivery of exosomes to specific tissues (18, 19). Luo et al. demonstrated that tumor-derived exosomes promote prostate cancer angiogenesis and metastasis by delivering phosphoglycerate mutase 1 (PGAM1), which binds γ-actin (ACTG1) (20). Exosomes also play a central role in CRPC progression. For instance, androgen deprivation therapy (ADT) alters exosomal miRNA profiles (e.g., upregulated miR-423-3p), driving the transition from androgen-dependent prostate cancer (ADPC) to CRPC via regulation of AR-V7 and ERG expression (21).

**Figure 1:**
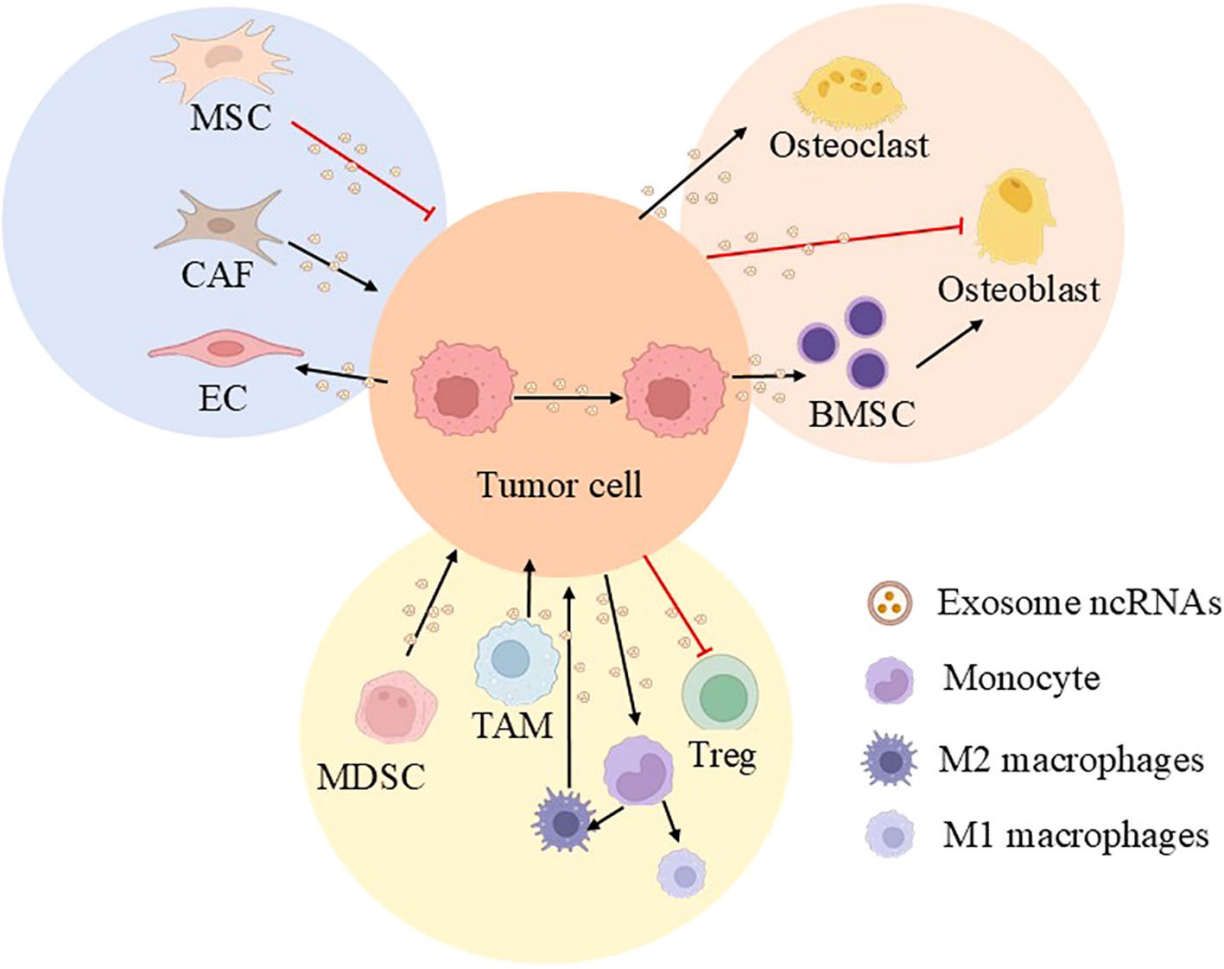
Outline of this review. This article takes the exosomal ncRNA communication in the tumor microenvironment of prostate cancer as the core starting point, systematically examining its functions in tumor-tumor, tumor-stromal, and tumor-immune cell crosstalk, as well as its pivotal role in shaping the bone metastatic niche. (MSC, mesenchymal stem cell; MDSC, myeloid-derived suppressor cell; BMSC, bone marrow mesenchymal stem cell; TAM, tumor-associated macrophage; CAF, cancer-associated fibroblast; Treg, regulatory T cell; EC, endothelial cell).

Evidence (22, 23) has shown that exosomes participate in the diagnosis and treatment of prostate cancer, acting as participants in pathophysiological mechanisms, biomarkers in cancer progression, and therapeutic tools. For instance, exosomes promote the progression of prostate cancer by carrying signaling molecules to act on tumor cells. However, growing recognition of ncRNAs has spurred extensive research into the functional roles of exosomal ncRNAs in prostate cancer. **Table 1** summarizes the mechanisms by which exosomal ncRNAs regulate prostate cancer progression. As evidenced by the compiled data, exosomal ncRNAs drive prostate cancer progression via diverse mechanisms, including competing endogenous RNA (ceRNA) networks and epigenetic regulation. Despite considerable advances in exosomal ncRNA research, current studies remain predominantly centered on modulating prostate cancer cellular phenotypes (33). Critical gaps persist in understanding their sorting mechanisms, spatiotemporal heterogeneity, and clinical translation challenges. Two prominent examples include the paucity of longitudinal data on dynamic changes in exosomal ncRNA expression throughout disease progression, and the limited evidence for their clinical utility as interventional targets. Furthermore, the complexity of cell-cell communication networks within the TME necessitates stematic categorization to elucidate underlying principles and inform clinical strategies. This review synthesizes findings from the past five years to systematically map ncRNA-mediated cellular interaction networks within the prostate cancer TME. Specifically, it examines the role of exosomal ncRNAs in facilitating communication: (1) between tumor cells; (2) between tumor and stromal cells; (3) between tumor and immune cells; and (4) in shaping the microenvironment of prostate cancer bone metastases (**Figure 1**). Our objective is to identify novel therapeutic targets and early diagnostic biomarkers while advancing combinatorial treatment approaches. Deciphering the regulatory signaling logic across cell types of exosomal ncRNAs may accelerate biomarker discovery and enable ncRNA-based therapeutic interventions, advancing the long-term goal of precision oncology for advanced prostate cancer.

**Table 1:** Overview of exosomal ncRNAs in prostate cancer.

Exosomal ncRNAs	Expression in PCa	Function	Target ncRNAs	Target genes	Reference
circTFDP2	Up	Proliferation (+) Migration (+)	/	PARP1	(24)
lncHOXD-AS1	Up	Invasion (+) Migration (+)	/	FOXM1	(25)
lncA1BG-AS1	Down	Proliferation (+) Migration (+) Invasion (+)	miR-361-5p	ZC3H13(m^6^A)	(26)
miR-423-5p	Up	Proliferation (+) Migration (+) Invasion (+) Apoptosis (−)	/	FRMD3	(27)
circ_0081234	Up	Migration (+) Invasion (+) EMT (+)	miR-1	MAP3K1	(28)
circHIPK3	Up	Proliferation (+) Migration (+) Invasion (+)	miR-212	BMI-1	(29)
lincROR	Up	Resistance (+)	/	MYH9	(30)
lncLINC01213	Up	AD resistance (+)	/	Wnt	(31)
miR-184	Up	Angiogenesis (+)	/	not mentioned	(32)

EMT, epithelial-mesenchymal transition; AD, androgen; PAPR1, poly(ADP‐ribose) polymerase 1; FOXM1, forkhead box protein M1; ZC3H13, zinc finger CCCH-type containing 13; FRMD3, FERM domain containing 3; MAP3K1, mitogen-activated protein kinase kinase 1; BMI-1, B-cell-specific moloney murine leukemia virus insertion site 1; MYH9, non-muscle myosin heavy chain IIA;/, exosomal ncRNAs do not regulate signaling pathways via the molecular sponge mechanism on ncRNAs.

## 2 Exosomal ncRNA-mediated crosstalk between tumor cells

Acting as key messengers in intercellular communication, exosomes play a multifaceted role in prostate cancer progression. This section examines the mechanisms by which exosomal ncRNAs mediate cellular crosstalk within the tumor microenvironment, promote tumor heterogeneity, and disseminate chemoresistance. Furthermore, it provides an in-depth exploration of their pivotal role in the development of CRPC and associated therapy resistance.

### 2.1 Exosome-mediated tumor cell–tumor cell signaling

The TME facilitates interactions between tumor cells and neighboring non-target bystander cells, generating biological phenomena termed the paracrine-like oncogenic influence (34, 35). This confirms that in the TME, interactions exist among tumor cells, further promoting tumor maintenance and development. These intercellular interactions can be achieved through direct cell contact or indirectly via secreted factors, and ncRNAs carried by exosomes play a crucial role in this process (36). Dai et al. (37) demonstrated that exosomal miR-183, upregulated in prostate cancer, enhances proliferation, invasion, and migration of LNCaP cells. Similarly, prostate cancer-derived exosomes transport circRNAs to exert analogous oncogenic effects. For instance, exosomal circKDM4A acts as a molecular sponge for miR-338-3p, alleviating its suppression of CUL4B to activate the ubiquitin-proteasome system, thereby fostering tumor proliferation and therapy resistance (38). Mechanical cues, including fluid shear stress, induce prostate cancer cells to release exosomes enriched with miR-21-5p (39). Uptake of these exosomes by adjacent cancer cells modulates migration-associated signaling pathways, altering cellular morphology and motility. These findings underscore the role of exosomal crosstalk in the TME and elucidate a novel mechanism whereby mechanical cues regulate metastasis via exosomal miRNA signaling.

### 2.2 Role of exosomes in driving intratumoral heterogeneity

Even within the same tumor, cancer cells exhibit heterogeneity in morphology, invasiveness, and responses to hormones or therapies, a phenomenon termed intratumoral heterogeneity (40, 41). This heterogeneity drives tumor progression and therapeutic failure (42). However, with the continuous deepening of research, researchers have found that during the process of tumor evolution, there are interactions among these heterogeneous cells. Often, high-grade tumor cells transfer malignant phenotypes (e.g., increased invasiveness, therapy resistance) to low-grade tumor subpopulations via exosomal cargo, enhancing their abilities in proliferation, invasion, and drug resistance. For example, exosomes derived from high-Gleason-score prostate cancer cells deliver miR-153 to adjacent cells, which modulates genes involved in cell cycle regulation and migration, thereby altering recipient cell behavior (43). Exosomal miR-150-5p was also significantly correlated with high Gleason scores and implicated in metastatic progression (44). Similarly, invasive prostate cancer cells secrete elevated levels of exosomal miR-424, which promotes tumor growth and metastasis by targeting downstream cell cycle regulators (45). In CRPC, plasma exosomes contain high levels of miR-222-3p and miR-375, which activate mTORC1 and STAT3 signaling via specific gene targets, enhancing cell proliferation and anti-apoptotic potential (46, 47).

### 2.3 Exosomal mechanisms in chemoresistance

Exosomal ncRNAs also mediate horizontal drug resistance transfer. Drug-resistant cells release FTH1P8, a pseudogene-derived RNA molecule, that binds ferritin heavy chain (FTH1) to reduce intracellular labile iron, inhibiting ferroptosis induction and spreading resistance to naive cells (48). Furthermore, researchers have found that a variety of circRNAs contribute to docetaxel resistance: circ-SFMBT2 acts as a miR-136-5p sponge to derepress TRIB1 and activate MAPK signaling (49); circ-XIAP upregulates TPD52 via miR-1182 sponging, inhibiting autophagy (50); and circSLC4A7 relieves miR-1205-mediated suppression of MAPT, enhancing microtubule stability (51). Collectively, these findings highlight exosomal communication as a critical driver of tumor heterogeneity and identify novel targets for overcoming docetaxel resistance.

### 2.4 Exosomal communication in ADT resistance and CRPC progression

**Figure 2:**
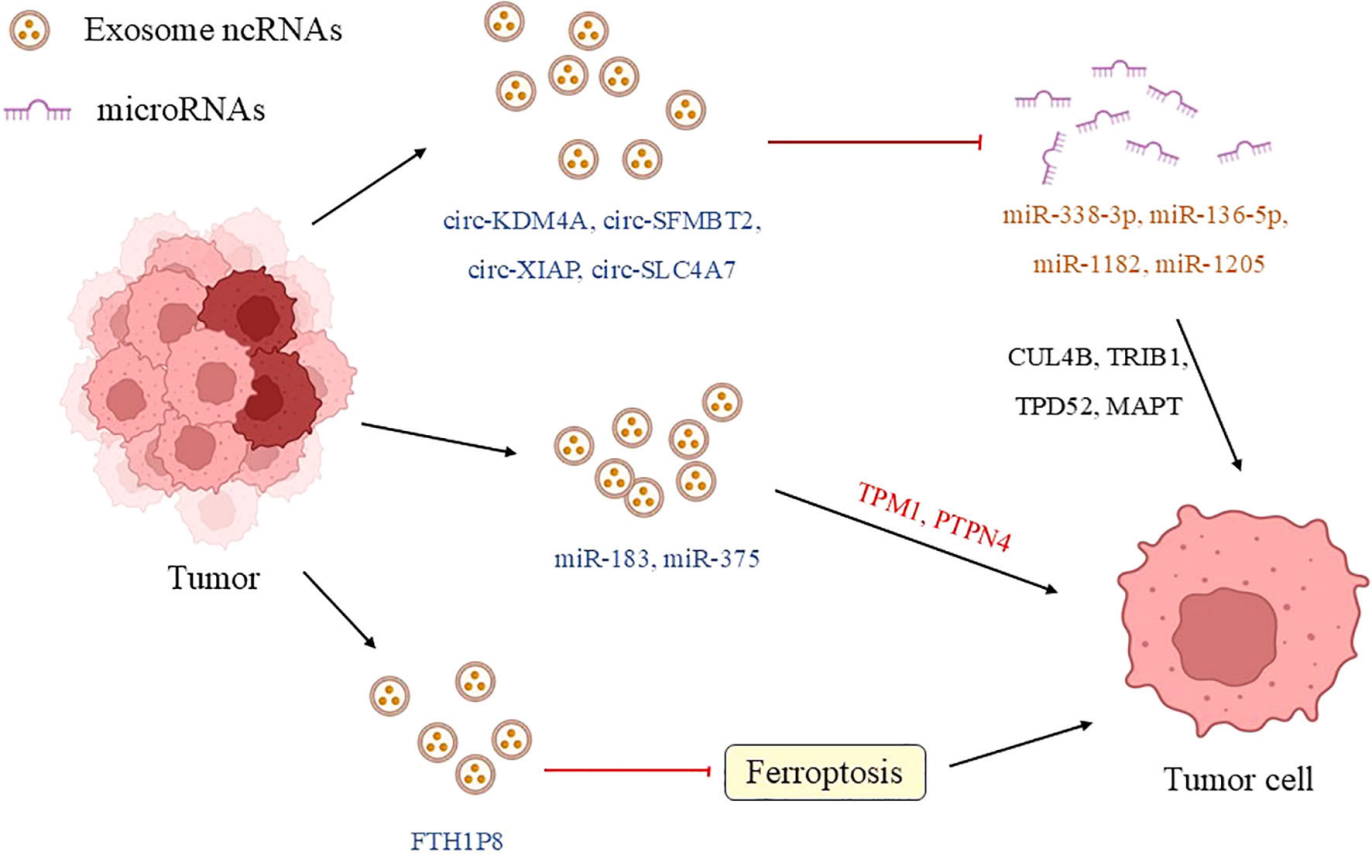
Exosomal ncRNA communication between tumor cells. In the tumor microenvironment of prostate cancer, tumor cells transfer ncRNAs (such as miR-183 and circKDM4A) through exosomes to regulate the biological behaviors of neighboring cells, promoting proliferation, invasion, and drug resistance, as well as tumor evolution and treatment resistance. For example, highly invasive cancer cells reprogram low-grade cells via exosomal miR-153/150-5p/424, while drug-resistant cells transmit chemotherapy resistance through exosomal circRNAs (such as circ-SFMBT2/XIAP/SLC4A7) or the pseudogene FTH1P8. These mechanisms reveal the central role of exosomal ncRNAs in the progression and drug resistance of prostate cancer. (TPM1, tropomyosin 1; PTPN4, phosphatase nonreceptor type 4; CUL4B, cullin 4B; STAT3, signal transducer and activator of transcription 3; TRIB1, tribbles homolog 1; TPD52, tumor protein D52; MAPT, microtubule-associated protein tau).

In addition to chemotherapeutic resistance, ADT resistance in prostate cancer represents a critical challenge, as the development of CRPC signifies progression to a more aggressive and refractory stage (52). Elucidating CRPC pathogenesis and identifying predictive biomarkers remain central objectives in current research. A recent study (53) demonstrated that during ADT-induced transition from androgen-dependent to androgen-independent prostate cancer, exosome secretion and cargo composition undergo significant alterations. Androgens were further shown to modulate exosomal miRNA expression profiles and surface protein levels, thereby altering exosome targeting specificity and intercellular communication dynamics. This discovery uncovered a novel ADT resistance mechanism: exosomal ncRNA-mediated cell communication. Subsequent investigations corroborated the pivotal role of exosomal ncRNAs in CRPC progression. Both previous prospective cohort studies and plasma exosomal miRNA sequencing results have shown that miR-423-3p is significantly upregulated in CRPC patients, suggesting its important role in CRPC (54). More direct evidence (46, 55) indicated that prostate cancer-derived exosomes transfer miR-222-3p and let-7a-5p between cells. By modulating AR-V7 and ERG expression, these exosomal miRNAs facilitate the transdifferentiation of ADPC cells into androgen-independent prostate cancer (AIPC)-like cells, thereby driving androgen resistance development (summarized in **Figure 2**).

## 3 Exosomal ncRNA communication in tumor-stromal cell interactions

The non-malignant components within the tumor microenvironment, along with their interacting molecular networks, are collectively termed the stroma of the tumor microenvironment (16). Functionally, these stromal components not only provide support for tumor cells but also act as pivotal regulatory hubs for modulating the biological behaviors of tumors, thereby having a profound influence on tumor progression (56, 57). The stroma comprises non-cellular components with the extracellular matrix as the central element, along with cellular components including CAFs, immune cells (such as tumor-associated macrophages and lymphocytes), endothelial cells, and mesenchymal stem cells (58–60). Mounting evidence (61–63) suggests that tumor cells and the stroma within the tumor microenvironment mutually regulate the biological behaviors of tumors, including proliferation, invasion, metastasis, immune escape, and treatment resistance, via intricate bidirectional interactions. In this context, we primarily focus on the communication mediated by exosomal ncRNAs between tumor cells and stromal cells.

### 3.1 Exosomal ncRNA communication with CAFs

CAFs constitute a heterogeneous population of activated fibroblasts predominantly located within the tumor stroma and represent one of the most abundant stromal cell types in the tumor microenvironment (64, 65). A recent text mining study (66) analyzing CAFs revealed that the research emphasis on CAFs has transitioned from their basic biological properties in the early research phase to their functional versatility, including pro-fibrotic activity, metabolic regulation, and extracellular matrix remodeling, as well as their interactions with other constituents of the tumor microenvironment, such as immune cells and exosomes. Analogously, numerous investigations have explored the interactions among CAFs, exosomes, and tumor cells in prostate cancer. These investigations have further delineated the molecular mechanisms underlying chemotherapy and castration resistance. For instance, miR-423-5p has been shown to promote chemotherapy resistance in tumor cells by targeting the TGF-β/Smad and ferroptosis pathways (67, 68). These findings not only propose a novel therapeutic target for countering drug resistance but also imply that detecting exosomal miR-423-5p could serve as a prognostic biomarker.

Furthermore, CAFs facilitate the adaptation of prostate cancer cells to adverse conditions, including androgen deprivation and hypoxia, by downregulating exosomal ncRNA miR-146a-5p (69). Another study (70) revealed that CAFs can secrete specific miRNAs, including miR-154 and miR-376c, and transfer them to tumor cells through exosomes. These miRNAs specifically target and suppress the expression of NKX3-1, leading to the dysregulation of the AR signaling pathway and driving the transformation of prostate cancer cells towards castration resistance. Under hypoxic microenvironments, CAFs can secrete exosomal miRNAs, including miR-500a-3p. MiR-500a-3p stabilizes heat shock factor 1 (HSF1) by inhibiting the E3 ubiquitin ligase FBXW7, thereby promoting the metastasis of prostate cancer cells in response to hypoxic stress (71). These studies collectively highlight the facilitatory role of CAFs in promoting the progression of prostate cancer cells via exosomal communication. They provide novel mechanistic insights into the resistance of prostate cancer to chemotherapy and androgen deprivation therapy and offer new perspectives for developing strategies to overcome tumor drug resistance.

### 3.2 Exosomal ncRNA communication with MSCs

Besides CAFs, mesenchymal stem cells (MSCs) represent another crucial cell type within the tumor microenvironment stroma. MSCs are a subtype of adult stem cells exhibiting multi-directional differentiation potential. They can be sourced from diverse tissues, including bone marrow, adipose tissue, and umbilical cord (72). Additionally, they are attracted to the tumor microenvironment by chemokines, such as cytokines and growth factors, secreted by tumors (73). MSCs exhibit a dual function in relation to tumor cells. On one hand, they can facilitate tumorigenesis and progression through mechanisms such as immune suppression, angiogenesis promotion, and stromal remodeling. For instance, gastric cancer cell-derived small extracellular vesicles can induce metabolic reprogramming of bone marrow-derived MSCs (BM-MSCs) via the ERK-PPARγ-CPT1A signaling pathway, thereby enhancing lymphatic metastasis potential (74–76). On the other hand, MSCs can exert an anti-tumor effect by inducing tumor cell apoptosis, inhibiting cell proliferation, or secreting anti-angiogenic factors, such as thrombospondin-1 (TSP-1) (77).

Recent investigations have demonstrated that in prostate cancer, MSCs can exert an anti-tumor function by secreting exosomes that shuttle ncRNAs to tumor cells. For instance, exosomal miR-187 originating from bone marrow-derived mesenchymal stem cells has been verified to infiltrate prostate cancer cells. It inhibits the proliferation, migration, and invasion of prostate cancer cells and promotes apoptosis by targeting the CD276/JAK3-STAT3-Slug axis (78). Analogously, another study (79) revealed that bone marrow-derived mesenchymal stem cells can transfer miR-99b-5p to prostate cancer cells through exosomes. This action inhibits the IGF1R signaling pathway, subsequently reducing tumor growth and decreasing drug resistance. The results of these studies are highly promising. The differential expression of exosomal ncRNAs, identified through gene chip sequencing, suggests that these expression profiles hold potential as diagnostic or prognostic biomarkers. Furthermore, MSCs, which are key components of the tumor microenvironment, represent potential therapeutic targets. For instance, engineered MSCs can be utilized as drug delivery vehicles to enhance therapeutic efficacy by targeting tumor sites and releasing anti-tumor agents. As reported in the study by Kurniawati et al. (80), exosomes derived from mesenchymal stem cells were utilized as a delivery vehicle for let-7c to specifically target the MYC and AKT2 signaling pathways in castration-resistant prostate cancer cells. The results indicated that the exosomes encapsulating let-7c decreased the tumor volume by 62% and abrogated enzalutamide resistance. This study capitalizes on the natural targeting capabilities of stem cells and exosomes to offer an effective and targeted tumor treatment strategy.

### 3.3 Exosomal ncRNA communication with endothelial cells

**Figure 3:**
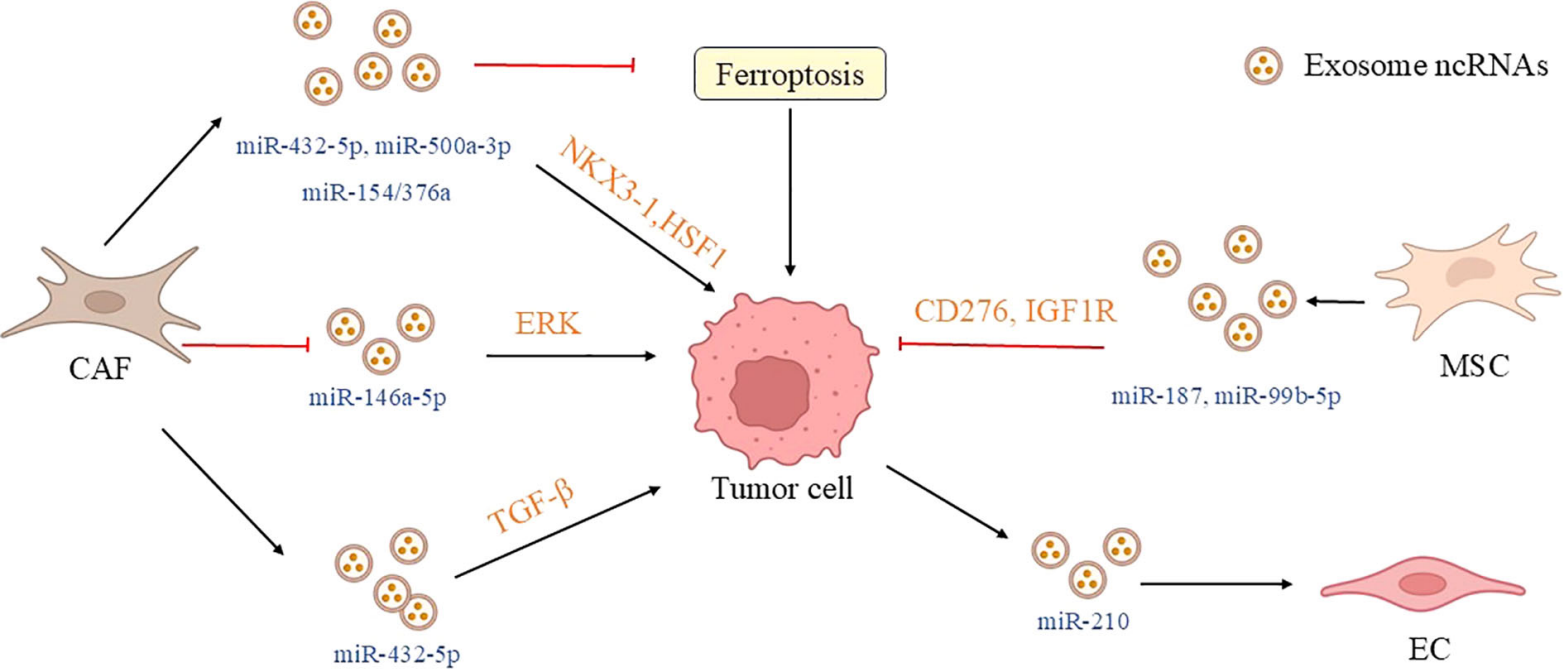
Exosomal ncRNA communication between tumor cells and stromal cells in the TME. Stromal components (such as CAFs, MSCs, and endothelial cells) in the tumor microenvironment form a bidirectional communication network with prostate cancer cells through exosomal ncRNAs, regulating tumor progression and treatment resistance. Exosomal miR-423-5p/miR-432-5p secreted by CAFs promotes chemoresistance and metastasis by targeting GREM2/CHAC1. The absence of CAF exosomal miR-500a-3p/miR-146a-5p induced by hypoxia or ADT enhances tumor adaptability. Exosomal miR-187/miR-99b-5p derived from MSCs can inhibit tumor growth. In addition, endothelial cells promote angiogenesis through exosomal miR-210, collectively forming a key regulatory network of the tumor microenvironment and providing a new strategy for intervening in prostate cancer by targeting stromal exosomes. (CAF, cancer-associated fibroblasts; MSC, mesenchymal stem cell; EC, endothelial cell; GREM2, gremlin-2; CHAC1, ChaC glutathione specific gamma-glutamylcyclotransferase 1; EGFR, epidermal growth factor receptor; ERK, extracellular regulated protein kinases; NKX3-1, NK3 homeobox 1; FBXW7, F-box and WD repeat domain-containing 7; HSF1, heat shock transcription factor 1; IGF1R, Insulin-like growth factor 1 receptor).

It is worth noting that endothelial cells, another important cell type in the tumor stroma of prostate cancer, have also been found to have communication via exosomal ncRNAs with tumor cells. Endothelial cells within the tumor microenvironment contribute to tumor progression beyond their well-established role in supporting angiogenesis and nutrient supply. These cells engage in signal transduction with cancer cells via the secretion of various growth factors. For example, they can upregulate immune checkpoint molecules such as PD-L1, fostering an immunosuppressive microenvironment that impairs the anti-tumor immune response (81). Recent studies have further elucidated that endothelial cells function as critical hubs for the communication of extracellular vesicles, including exosomes. Through the reception and release of bioactive molecules, they significantly extend their regulatory influence over tumor biological behavior. *In vitro* experiments have found that exosomes secreted by PC-3 cells can promote the proliferation, migration, and lumen formation of endothelial cells. Further research (82) has confirmed that PC-3 exosomes are rich in certain pro-angiogenic proteins and microRNAs (such as miR-210). These molecules can promote angiogenesis by activating the relevant signaling pathways within endothelial cells (summarized in **Figure 3**). This result not only provides a new perspective for a deeper understanding of the angiogenesis mechanism of prostate cancer but also offers potential targets and strategies for the treatment of prostate cancer, suggesting that interventions targeting PC-3 exosomes and their related signaling pathways may help inhibit the angiogenesis and tumor growth of prostate cancer.

## 4 Exosome-derived ncRNAs modulate tumor-immune cell dialogues

The role of the immune response in tumorigenesis should not be underestimated. Consequently, the tumor immune microenvironment (TIME), a critical component of the TME, has attracted significant research interest. TIME centers on immune regulation within the TME, encompassing immune cells (e.g., T cells, myeloid-derived suppressor cells (MDSCs), tumor-associated macrophages (TAMs)), immune checkpoint molecules (e.g., PD-1/PD-L1), cytokines (e.g., IL-6, TGF-β), and their interactive networks (83, 84).

### 4.1 Immune cells in TIME

Immune cells within the TME constitute the fundamental components of the tumor immune ecosystem. These immune cells exhibit not only remarkable diversity in cell types but also intricate complexity in their functions, being capable of either suppressing tumor progression or enabling tumor immune escape. For example, during the early tumorigenic stages, immune cells such as CD8^+^ T cells and natural killer (NK) cells exert anti-tumor effects via immune surveillance (85). In contrast, MDSCs and M2-polarized macrophages secrete a repertoire of cytokines that establish an immunosuppressive microenvironment conducive to tumor progression (86, 87). Notably, a bidirectional regulatory relationship exists between tumor cells and immune cells. Tumor cells can establish an immunosuppressive microenvironment and evade immune surveillance by modulating immune cells. For instance, tumor cells can upregulate glycolysis (the Warburg effect), thereby competing for glucose in the microenvironment and impairing T cell function. Simultaneously, tumor cells can hinder the maturation of dendritic cells via lipid metabolites, such as prostaglandin E2 (88). Moreover, tumors can attract TAMs and MDSCs by secreting factors including vascular endothelial growth factor (VEGF) and colony-stimulating factor 1 (CSF-1). These recruited cells subsequently secrete enzymes, such as arginase 1 (Arg1) and inducible nitric oxide synthase (iNOS), which consume arginine crucial for T cell activation and generate immunosuppressive metabolites (89). In summary, the TIME represents a complex ecosystem comprising tumor cells, immune cells, diverse cytokines, and metabolites within tumor tissues. Its dynamic equilibrium directly influences tumor initiation, progression, metastasis, and the response to therapy, positioning it as one of the central areas of current tumor research.

### 4.2 Overview of the prostate cancer TIME

Unlike most tumors, the TIME of prostate cancer is predominantly immunosuppressive. Even at advanced tumor stages, the immunosuppressive microenvironment persists (90, 91). Specifically, it exhibits low PD-L1 expression, sparse immune cell infiltration (e.g., reduced CD8^+^ T cell abundance), and an enrichment of inhibitory immune cell populations, such as TAMs, MDSCs, and regulatory T cells (Tregs) (92). Moreover, the secretion of inhibitory cytokines, including transforming growth factor-β (TGF-β), interleukin-8 (IL-8), and interleukin-10 (IL-10), further intensifies immunosuppression (93). When compared to other solid tumors, like bladder cancer, prostate cancer represents a typical “immune-cold tumor.” In both the primary tumor site and in CRPC, immune cell infiltration is minimal. Likewise, in metastatic prostate cancer, particularly in bone metastases, the immune microenvironment exhibits enhanced immunosuppression, marked by an enrichment of M2-polarized macrophages and T-cell exhaustion (94, 95). This results in a low response rate to immune checkpoint inhibitors, such as PD-1/PD-L1 antibodies (96, 97). Additionally, recent studies have demonstrated that the androgen receptor (AR) signaling pathway plays a pivotal role in prostate cancer and engages in complex interactions with the immune microenvironment. AR signaling not only promotes the growth of prostate cancer epithelial cells but also directly regulates the establishment of the immune microenvironment by inhibiting antigen presentation (e.g., major histocompatibility complex class I (MHC-I) expression) and facilitating the recruitment of immunosuppressive cells (e.g., MDSCs) (98). Another study (99) has indicated that ADT can induce dynamic alterations in the composition of immune cells within the tumor microenvironment. Long-term ADT can activate immunosuppressive factors, such as interferon alpha 17 (IFNA17), contributing to the formation of a drug-resistant microenvironment, although the underlying mechanism remains incompletely understood. These immune features of prostate cancer present significant challenges to current immunotherapy strategies for prostate tumors.

Thus, delving deeper into the mechanisms underlying cell functions within the immune microenvironment of prostate cancer holds substantial potential for surmounting this challenge. Subsequently, we summarize the communication mediated by exosomal ncRNAs between tumor cells and immune cells within the tumor microenvironment, aiming to further elucidate the cell communication network in the immune microenvironment of prostate cancer.

### 4.3 Immunostimulatory exosomal ncRNA communication

As previously discussed, the tumor immune microenvironment displays temporal heterogeneity. During the early phases of tumorigenesis, the immune microenvironment predominantly exerts a tumor-suppressive effect. Nevertheless, as the tumor advances, a tumor-immunosuppressive microenvironment gradually emerges. We hypothesized that this might be partially attributable to the exosomal communication between tumor cells and immune cells. In the prostate cancer tumor microenvironment, exosomes function as critical mediators of intercellular communication, regulating complex immune responses through the delivery of bioactive molecules. Although macrophage polarization is a key aspect of this regulation, the influence of exosomes on other immune cells warrants further investigation. Research (100) demonstrates that exosomes secreted by prostate cancer cells are enriched with the chemokine CXCL14. Upon uptake by macrophages, these exosomes trigger NF-κB signaling to induce M2 polarization, thereby establishing an immunosuppressive microenvironment that facilitates tumor progression. Similarly, exosomes derived from PC3 cells promote M2 macrophage polarization via the delivery of miR-let-7b (101). Furthermore, recent studies (102) indicate that YY1 expression in M2 macrophages is upregulated by super-enhancers, promoting the secretion of exosomes containing circ-0000326. Following their uptake by prostate cancer cells, these exosomes elevate FZD7 expression by sponging miR-1258, which activates the Wnt/β-catenin signaling pathway and forms a positive feedback loop. Beyond macrophages, other immune cells significantly contribute to prostate cancer progression. For instance, exosomes from MDSCs advance CRPC through the S100A9/circMID1/miR-506-3p/MID1 axis. Mechanistically, exosomal S100A9 upregulates circMID1, which then sequesters miR-506-3p, alleviating its suppression of MID1 and subsequently activating the mTOR signaling pathway. Notably, inhibiting S100A9 significantly attenuates the tumor-promoting effects of these exosomes, reducing tumor growth by 51% (103). Meanwhile, miR-95 within TAM-derived exosomes drives tumor malignancy through multiple mechanisms. It directly targets the JUNB gene to enhance proliferation, invasion, and epithelial-mesenchymal transition in prostate cancer cells. Concurrently, miR-95 impairs endocytosis by suppressing SNX1 expression, thereby amplifying cellular sensitivity to growth factors like EGF and promoting migration and angiogenesis. Clinical analyses confirm that elevated miR-95 expression correlates with adverse clinicopathological features (104).

### 4.4 Immunosuppressive exosomal ncRNA communication

**Figure 4:**
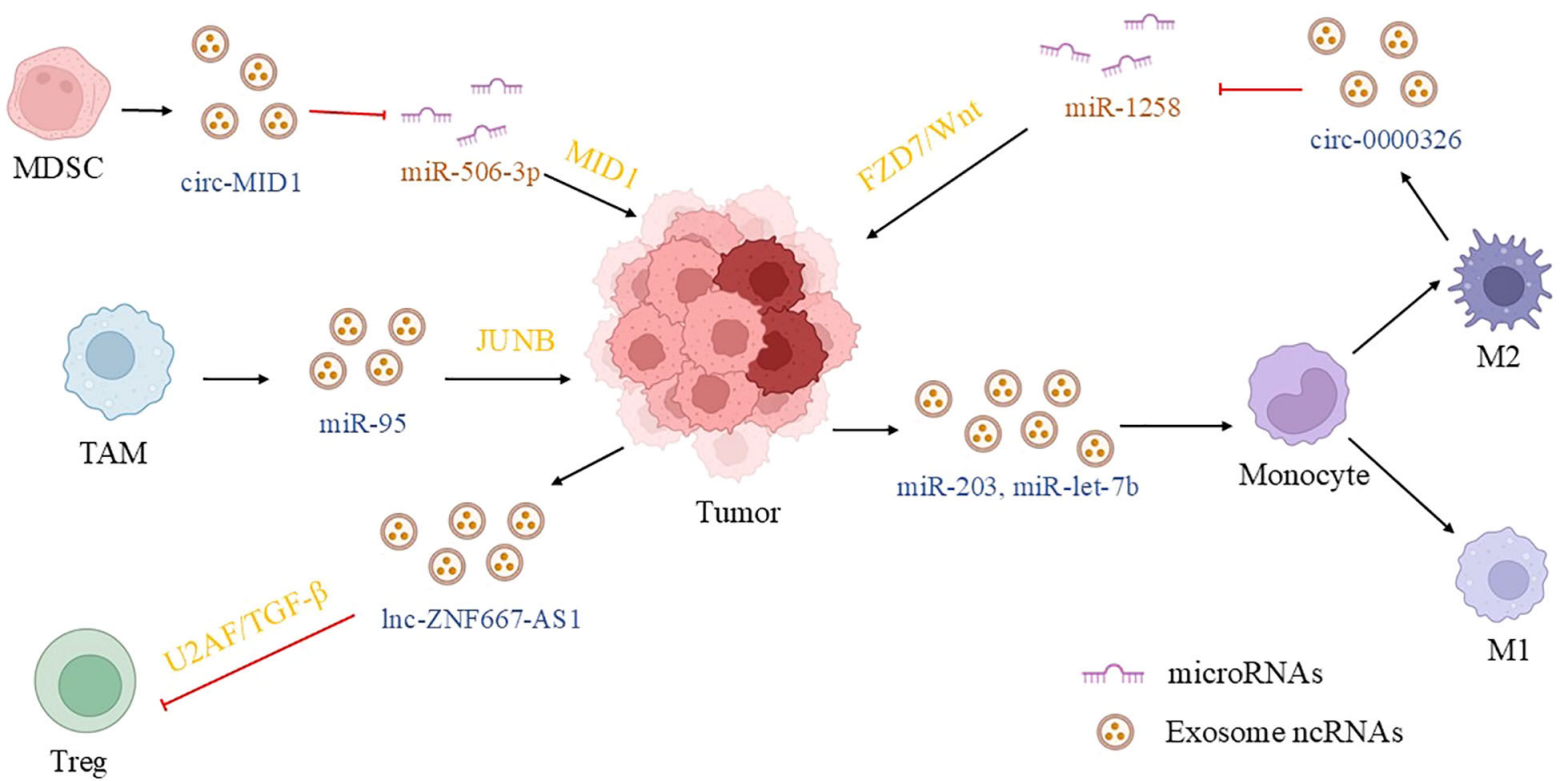
Exosomal ncRNA communication between prostate cancer cells and immune cells in the TME. In the immune microenvironment of prostate cancer, tumor cells and immune cells form a bidirectional regulatory network through exosomal ncRNAs. Exosomal CXCL14/miR-let-7b secreted by tumor cells induces the polarization of M2 macrophages, and M2 macrophages activate the Wnt pathway through exosomal circ-0000326 to promote tumor progression. Meanwhile, exosomal ZNF667-AS1/miR-203 of prostate cancer cells can inhibit Treg cells or induce M1 macrophages, enhancing anti-tumor immunity. In addition, immune cells (such as MDSCs/TAMs) directly promote tumor proliferation and EMT through exosomal circMID1/miR-95. This dynamic balance not only maintains the immunosuppressive microenvironment but also has the potential for immune activation, providing new strategies for targeting the immune microenvironment. (MDSC, myeloid-derived suppressor cells; TAM, tumor associated macrophage; M2, M2 macrophages; M1, M1 macrophages; MID1, midline1; JUNB, Jun B proto-oncogene; U2AF, U2 small nuclear RNA auxiliary factor 1; FZD7, frizzled class receptor 7).

Regarding tumor-suppressive mechanisms, exosomal ncRNAs employ diverse regulatory pathways. For instance, the lncRNA ZNF667-AS1, derived from prostate cancer cells and delivered via exosomes, binds to the U2AF1 protein. This interaction impairs the stability of transforming growth factor-β receptor 1 (TGFBR1) mRNA, resulting in decreased TGFBR1 expression. This downregulation not only inhibits the expansion of Tregs, thereby attenuating the immunosuppressive microenvironment, but also significantly enhances tumor cell chemosensitivity to docetaxel. Animal experiments confirmed that the administration of exosomes carrying ZNF667-AS1 increased chemosensitivity by 2.3-fold (105). Separately, exosomal miR-203 secreted by prostate cancer cells induces macrophage polarization toward the M1 phenotype by suppressing SOCS3 expression and subsequently activating the STAT1 signaling pathway. The polarized M1 macrophages secrete cytokines, including tumor necrosis factor-α and interleukin-12, which directly inhibit tumor cell proliferation and migration while inducing apoptosis. In a mouse model, miR-203 overexpression reduced tumor volume by 58%, demonstrating its therapeutic potential to reverse the immunosuppressive microenvironment through macrophage reprogramming (106). Collectively, these studies reveal a complex, exosome-mediated regulatory network within the prostate cancer tumor microenvironment. On one hand, immune cells such as MDSCs and TAMs accelerate tumor progression by delivering oncogenic molecules like circMID1 and miR-95. Conversely, exosomes derived from tumor cells can also carry tumor-suppressive molecules such as ZNF667-AS1 and miR-203, which inhibit tumor growth by modulating Treg expansion, promoting anti-tumor macrophage polarization, and enhancing chemosensitivity. (summarized in **Figure 4**). These studies further elucidate the mechanisms underlying prostate cancer progression and enhance our understanding of the prostate cancer tumor immune microenvironment.

## 5 Exosomal ncRNA signaling in remodeling the bone metastatic tumor microenvironment

### 5.1 Overview of PCa bone metastasis biology

Bone metastasis in advanced prostate cancer is one of the primary causes of death among patients, and this metastatic process is governed by intricate molecular mechanisms (107). Generally, this metastatic process encompasses several key steps, including primary tumor invasion, EMT, the survival and homing of circulating tumor cells, colonization within the bone microenvironment, and the bidirectional regulation between osteolysis and osteogenesis (108). Prostate cancer cells gain migratory capacity via EMT, detach from the primary tumor locus, and subsequently enter the circulatory system. Subsequently, these cells migrate through the bloodstream and lymphatic vessels. Guided by chemokines, they home in a directional manner to the bone marrow microenvironment (109, 110). Notably, certain tumor cells that have colonized within the bone metastasis microenvironment may enter a dormant state and can be reactivated subsequently upon stimulation by environmental factors, such as inflammatory factors (111, 112). Osteoblasts, osteoclasts, and mesenchymal stem cells within the bone marrow secrete growth factors, thereby creating a microenvironment conducive to tumor growth (113). Within this bone metastasis microenvironment, cancer cells activate osteoblasts and osteoclasts through the secretion of diverse factors, leading to the induction of osteoblastic and osteoclastic lesions. In prostate cancer, the lesions are predominantly osteoblastic, with accompanying osteolytic activities. Conversely, the activated osteoblasts and osteoclasts secrete growth factors, which in turn further stimulate tumor growth, thus establishing a vicious cycle (114). During the process of bone metastasis, osteoblasts secrete chemokines and growth factors, which serve as tumor homing signals and facilitate the adhesion of tumor cells to the tumor stroma (115, 116). Meanwhile, upon activation, osteoclasts induce bone resorption, releasing TGF-β, insulin-like growth factor-1 (IGF-1), calcium ions, and other substances stored in the bone matrix, thereby providing proliferation signals for tumor cells. Moreover, the cavities generated by osteoclast-mediated bone resorption offer physical space for tumor cell colonization (117). In summary, bone metastasis in prostate cancer is the outcome of dynamic interactions between cancer cells and the bone microenvironment, involving crucial processes such as EMT, chemokine-mediated homing, and the establishment of self-reinforcing feedback loops. Additionally, the bone metastasis process extensively entails interactions among diverse cell types, with tumor cells, osteoblasts, and osteoclasts being particularly significant. A more in-depth understanding of these mechanisms is instrumental in the development of targeted therapeutic strategies and the enhancement of patient prognosis. Future research endeavors should concentrate on tumor heterogeneity, the eradication of dormant cells, and the formulation of microenvironment reprogramming strategies.

As previously discussed, within the bone metastasis microenvironment, tumor cells, osteoblasts, and osteoclasts interact with one another, giving rise to a cyclic “vicious triangle”. This interaction not only perturbs bone homeostasis but also establishes a mutually reinforcing cycle through the release of diverse factors, thereby exacerbating disease progression. Consequently, an increasing number of studies are commencing to investigate the mechanisms underlying the interactions among these three cell types. In this section, we summarize the communication mediated by exosomal ncRNAs among these three cell types during the bone metastasis process of prostate cancer.

### 5.2 Tumor-derived exosomal ncRNAs affecting bone cells

Certain exosomal ncRNAs can activate osteogenic differentiation pathways, inducing a transition in the bone microenvironment towards an “osteoblastic” phenotype that provides structural support for tumor cell colonization. Among these, lncRNA NEAT1 and miR-375 are key regulatory factors. *In vitro* co-culture experiments have demonstrated that exosomes derived from prostate cancer cells are internalized by human bone marrow mesenchymal stem cells (hBMSCs). Subsequently, NEAT1 sequesters miR-205-5p, which relieves the suppression of RUNX2 and activates osteogenic differentiation signaling. This finding was further validated in nude mouse models, where exosomes overexpressing NEAT1 increased the number of mineralized nodules in bone tissue and promoted the formation of bone metastatic lesions (118). Similarly, *in vitro* experiments confirmed that exosomal miR-375 promotes the proliferation and migration of prostate cancer cells and induces the osteogenic differentiation of hBMSCs by targeting the DIP2C gene and consequently activating the Wnt/β-catenin pathway. Notably, serum exosomes from patients with bone metastasis exhibited significantly elevated miR-375 levels, and its expression positively correlated with bone scan positivity, suggesting its involvement in clinical bone metastasis progression (119).

In contrast to tumor-promoting ncRNAs, other exosomal ncRNAs inhibit osteoblast proliferation and differentiation by targeting key osteogenic genes, leading to diminished bone formation and even osteolytic lesions. miR-1275 and miR-940 are representative of this category, with the former’s inhibitory effect underscoring the signaling complexity within the bone microenvironment. *In vitro* osteoblast culture experiments revealed that upon uptake of prostate cancer cell-derived exosomal miR-1275, osteoblasts exhibit targeted silencing of the SIRT2 gene. SIRT2 downregulation reduces RUNX2 deacetylation, ultimately suppressing osteoblast proliferation and mineralization. Verification in a mouse tibial injection model confirmed that exosomes overexpressing miR-1275 reduce osteocalcin (OCN) expression in bone tissue and exacerbate osteolytic damage, providing a mechanistic explanation for the coexistence of osteolytic and osteoblastic lesions in clinical prostate cancer bone metastasis (120). Notably, exosomes isolated from a mouse orthotopic prostate cancer model were found to significantly impair osteoblast mineralization upon co-culture. This effect was mediated by exosomal miR-940, which suppresses the expression of key osteogenic differentiation genes, including ALP and RUNX2 (121). This study identifies one of the few inhibitory ncRNAs validated in *in vivo* models, presenting a stark contrast to the multitude of ncRNAs that promote tumor progression. This antagonistic relationship suggests that the bone microenvironment is governed by a dynamic balance between “pro-metastatic” and “anti-metastatic” signals, rather than being driven solely by promotional influences. This insight provides a new perspective for understanding the complex pathology of bone metastasis.

The disruption of osteoclast differentiation balance is a major contributor to bone damage in metastasis. Exosomal ncRNAs can influence osteoclastogenesis by modulating relevant differentiation pathways, with miR-92a-1-5p exerting a pro-osteoclast effect and miR-148a an anti-osteoclast effect. *In vitro* experiments on osteoclast precursor cells demonstrated that exosomal miR-92a-1-5p targets and inhibits the expression of the COL1A1 gene. This not only impairs osteogenic function but also activates osteoclastogenic transcription factors such as NFATc1, thereby promoting the fusion of precursor cells into mature, multinucleated osteoclasts (122). Conversely, following their uptake by osteoclast precursor cells, exosomes from prostate cancer PC-3 cells exert an anti-osteoclast effect via the downregulation of miR-148a. The reduction in miR-148a leads to the upregulation of its target gene, MAFB, and concurrently inhibits the PI3K/AKT/mTOR pathway, ultimately diminishing the formation of TRAP-positive multinucleated cells (123).

### 5.3 Bone cell-derived exosomal ncRNAs affecting tumor cells

Osteoblasts within the bone microenvironment do not passively receive tumor-derived signals; they actively contribute to tumor progression by secreting exosomes that deliver ncRNAs, which in turn enhance the proliferation, migration, and bone colonization capacity of prostate cancer cells. This process constitutes a critical link in the pathological synergy between tumors and bone. Current research has identified two core regulatory factors in this pathway: miR-140-3p and circ-DHPS, whose mechanisms have been validated through both *in vitro* and *in vivo* experiments. *In vitro* studies demonstrated that exosomes secreted by osteoblasts are internalized by prostate cancer cells (LNCaP, PC-3), subsequently promoting cell proliferation and migration by modulating the autophagic pathway. Subsequent validation in a nude mouse bone metastasis model confirmed that injection of osteoblast-derived exosomes significantly increased the burden of tumor cell colonization in bone tissue (124). In CRPC, a distinct clinical subtype, the role of osteoblast-derived exosomes is further modulated. A key study (125) revealed that AR inactivation alters the ncRNA cargo of osteoblast-derived exosomes, thereby influencing tumor cell chemotactic migration. Specifically, following AR knockdown in osteoblasts *in vitro*, the expression of circular RNA circ-DHPS in their exosomes was significantly upregulated. Upon internalization by prostate cancer cells, circ-DHPS acts as a molecular sponge for miR-214-3p. This sequestration relieves the miR-214-3p-mediated suppression of the chemokine CCL5, leading to enhanced CCL5 expression and consequently promoting tumor cell adhesion and invasion.

### 5.4 Reciprocal signaling via MSCs

As multipotent stem cells within the bone microenvironment, MSCs possess the capacity to differentiate into various lineages, including osteoblasts and adipocytes, and are integral to immune regulation and cytokine secretion. Exosomes derived from prostate cancer cells can indirectly influence bone metabolic balance and tumor progression by modulating MSC function. Key molecules mediating this indirect communication include lncAY927529 and miR-142-3p. *In vitro* experiments demonstrated that lncAY927529, carried by prostate cancer cell-derived exosomes, is internalized by ST2 cells (a model MSC line). By modulating autophagic activity in ST2 cells, lncAY927529 influences the secretion of the chemokine CXCL14, which in turn promotes the proliferation and invasion of neighboring prostate cancer cells via a paracrine mechanism (126). This pathway was validated using *in vitro* Transwell and co-culture models, indicating that lncRNAs can indirectly contribute to remodeling the bone metastatic niche by altering the secretory profile of MSCs. Furthermore, the regulation of MSCs by exosomal ncRNAs is susceptible to pharmacological intervention. As reported in study (127), propofol, a commonly used clinical anesthetic, can impair the osteogenic differentiation of MSCs by altering exosomal ncRNA levels. Propofol treatment significantly reduced exosomal miR-142-3p, concurrently inhibiting the osteogenic potential of MSCs. Mechanistic investigation revealed that miR-142-3p promotes osteogenesis by targeting and suppressing negative regulators of this process, such as DKK1. Propofol abrogates this pro-osteogenic effect by downregulating miR-142-3p, ultimately impeding the formation of a hospitable bone metastatic microenvironment.

### 5.5 Therapeutic and biomarker potential

The deepening understanding of regulatory mechanisms has revealed the considerable clinical potential of exosomal ncRNAs for the early diagnosis, prognostic assessment, and therapeutic targeting of prostate cancer bone metastasis. Current research has made preliminary progress in identifying diagnostic biomarkers and exploring therapeutic strategies. For instance, a comparative analysis of plasma exosomes from 82 patients with prostate cancer bone metastasis, 65 patients without bone metastasis, and 40 healthy controls identified a signature of four differentially expressed miRNAs (hsa-miR-125a-3p, hsa-miR-330-3p, hsa-miR-339-5p, hsa-miR-613). Among these, the expression level of hsa-miR-125a-3p was positively correlated with the stage of bone metastasis, demonstrating a diagnostic sensitivity of 78.1% and a specificity of 75.4% (128). Subsequent follow-up data indicated that patients with high hsa-miR-125a-3p expression had significantly shorter bone metastasis-free survival, suggesting its utility not only for early diagnosis but also as a prognostic indicator. Furthermore, analysis of clinical samples confirmed that serum exosomal miR-375 levels were significantly elevated in patients with bone metastasis compared to those without, and its expression positively correlated with the burden of bone metastatic lesions, thereby reinforcing the feasibility of exosomal miRNAs as diagnostic biomarkers (119).

**Figure 5:**
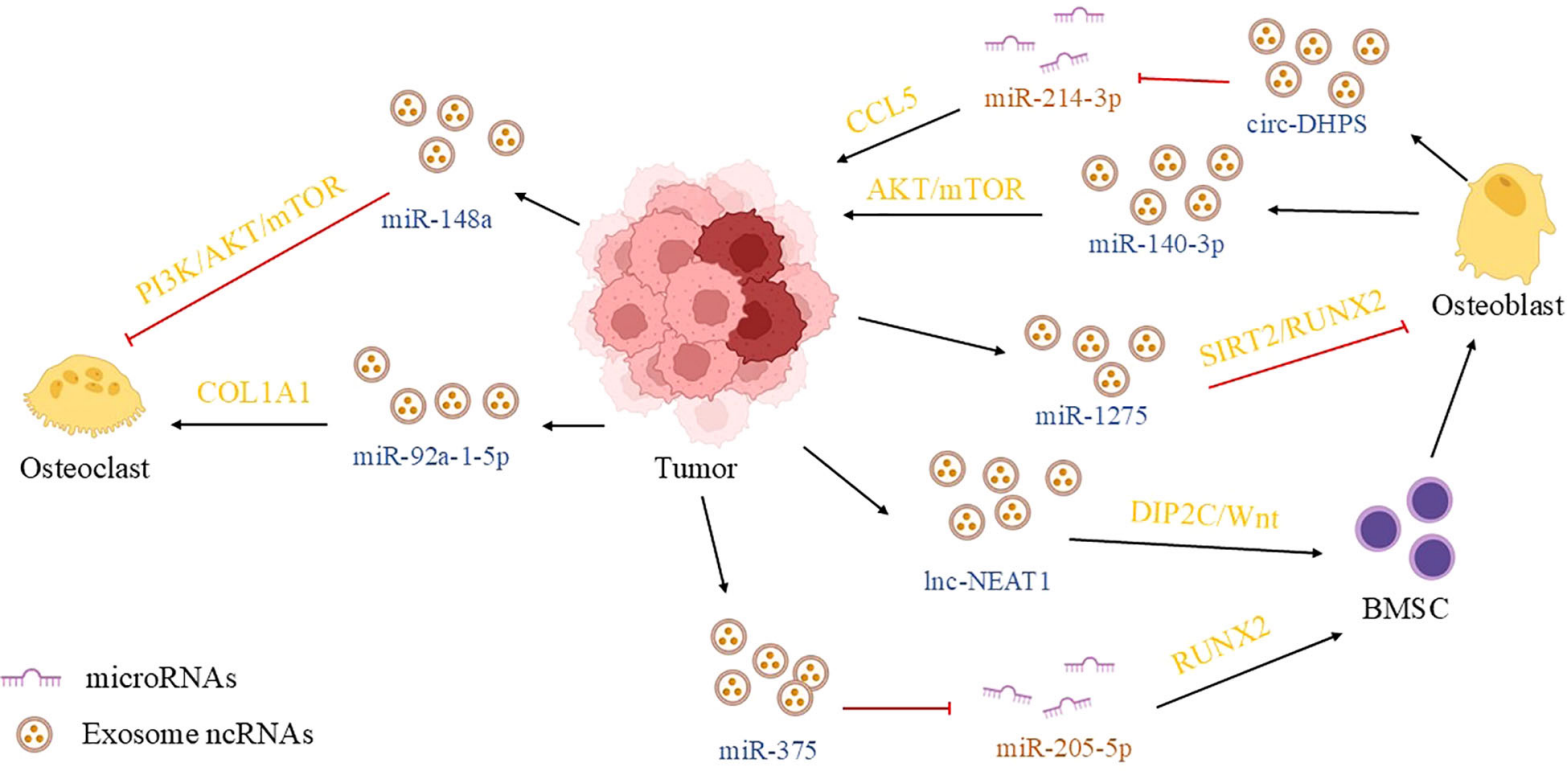
The role of exosomal ncRNA communication in shaping the bone metastasis microenvironment of prostate cancer. In the bone metastasis microenvironment of prostate cancer, tumor cells, osteoblasts, and osteoclasts form a “vicious triangle” through exosomal ncRNAs: PCa exosomes carry NEAT1/miR-375 to induce the osteogenic differentiation of mesenchymal stem cells, while miR-1275/miR-92a-1-5p inhibits osteogenic activity or activates osteoclasts, disrupting bone homeostasis. Osteoblasts promote tumor progression through exosomal miR-140-3p/circ-DHPS, and ncRNAs related to osteoclast activation (such as miR-92a-1-5p) exacerbate osteolytic lesions. This suggests that targeting exosomal communication in the bone microenvironment may become a new anti-metastasis strategy. (BMSC, bone marrow-derived mesenchymal stem cells; ACER2, alkaline ceramidase 2; CCL5, C-C chemokine ligand 5; SIRT2, sirtuin 2; RUNX2, runt-related transcription factor 2; DIP2C, disco interacting protein 2 homolog C).

Leveraging these elucidated regulatory mechanisms, targeting exosomal ncRNAs or their downstream pathways has emerged as a promising direction for treating bone metastasis. *In vitro* experiments demonstrated that inhibition of miR-1275 significantly restored SIRT2/RUNX2 signaling in osteoblasts and increased mineralized nodule formation (120). A separate study confirmed that knockdown of miR-375 suppressed the proliferation and migration of prostate cancer cells by attenuating Wnt pathway activity (119). Moreover, a combination strategy that concurrently targets key ncRNAs and critical pathway components within the tumor-bone microenvironment may yield synergistic therapeutic effects. For example, one investigation found that the combined application of a miR-140-3p inhibitor and an AKT inhibitor more potently suppressed the proliferation and bone colonization of prostate cancer cells (124). This combinatorial approach demonstrated robust anti-tumor efficacy in nude mouse models, providing a strong experimental foundation for future clinical translation. (summarized in **Figure 5**).

## 6 Conclusion and prospect

PCa, which ranks among the leading causes of cancer-related fatalities in men globally, continues to present significant clinical challenges regarding treatment resistance and metastatic spread. In recent years, exosomes, serving as crucial mediators of intercellular communication within the TME, have garnered substantial attention. This is because of the distinctive roles played by the ncRNAs they carry in regulating tumor progression, facilitating immune escape, and contributing to drug resistance.

Exosomal ncRNAs act as “molecular messengers” in the TME of PCa by mediating intercellular communication. In the intercellular communication between tumor cells, exosomal ncRNAs serve as molecular messengers, promoting tumor progression and enhancing drug resistance. For instance, prostate cancer cells with a high Gleason score secrete exosomal microRNAs (miR-153, miR-150-5p, and miR-424), which reprogram neighboring low-grade cells and enhance their invasive phenotypes (43–45). In addition, the bidirectional exosomal communication between tumor cells and stromal cells (such as CAFs and MSCs) further shapes the pro-tumor microenvironment (67, 68, 70, 71, 78, 79). On the other hand, the immune characteristics of the prostate cancer TIME are closely associated with the regulation of exosomal ncRNAs. Tumor cells induce the polarization of macrophages towards the M2 phenotype via exosomal CXCL14 or microRNA (miR-let-7b), suppressing the anti-tumor immune response (100, 101). Meanwhile, circular RNA (circMID1) or microRNA (miR-95) secreted by MDSCs or TAMs directly drives the progression of CRPC (103, 104). Intriguingly, some exosomal ncRNAs (such as ZNF667-AS1 or miR-203) can also counteract immunosuppression, augmenting the anti-tumor effect by inhibiting the proliferation of Tregs or inducing the polarization of M1 macrophages (105, 106). This bidirectional regulation unveils the intricate role of exosomal ncRNAs in the prostate cancer TIME, suggesting their potential as targets for enhancing immunotherapy sensitivity. Similarly, in the bone metastasis microenvironment, exosomal ncRNAs exacerbate the imbalance between osteogenesis and osteolysis by mediating the “vicious triangle” interaction (119, 122, 124, 125, 129). To summarize, exosomal ncRNAs collectively drive the malignant progression of prostate cancer by mediating complex intercellular crosstalk within TME. Then, how does this intricate communication network evolve as the disease progresses from local advancement to castration resistance, and eventually colonizes the bone? During tumor proliferation and early metastasis, exosomal miR-183 promotes cancer cell invasion and bone metastasis by targeting TPM1 (37), whereas circKDM4A upregulates CUL4B by sponging miR-338-3p to drive malignant progression (38). The progression to CRPC marks a critical turning point, characterized by distinct alterations in exosomal ncRNA expression: For instance, miR-222-3p and miR-375 enhance treatment resistance by activating the mTOR signaling pathway and the PTPN4/STAT3 axis, respectively (46, 47). Similarly, let-7a-5p and LINC01213 facilitate the transition to androgen-independent growth, thereby promoting the development of CRPC (31, 55). Furthermore, circRNAs including circ-SFMBT2, circSLC4A7, and circ-XIAP confer docetaxel resistance via ceRNA mechanisms, collectively forming a robust cross-resistance network (49–51). The role of exosomal ncRNAs in remodeling the TME to drive distant metastasis is also significant. For example, CAF-derived miR-500a-3p and miR-423-5p remodel the microenvironment to promote metastasis and drug resistance under hypoxic or post-treatment conditions (50, 68). Additionally, macrophage-associated circ-0000326 accelerates disease progression via the Wnt signaling pathway (102). Ultimately, in the terminal stage of bone metastasis, exosomal molecules such as miR-375 and lncRNA NEAT1 orchestrate osteoblastic and osteoclastic activity, thereby generating a pre-metastatic niche that supports tumor colonization (118, 119). Collectively, these findings delineate a detailed regulatory network of exosomal ncRNAs throughout the dynamic evolution of prostate cancer, thereby enhancing our understanding of its underlying mechanisms.

In addition, exosomal ncRNAs have increasingly been applied in clinical translational research. In the context of diagnosis, plasma exosomal microRNA (miR-423-3p) or microRNA combinations (such as miR-125a-3p, miR-613) have demonstrated potential as early diagnostic markers for CRPC or bone metastasis (21, 130). The stability and accessibility of ncRNAs render them ideal biomarkers for early diagnosis. For example, in lung cancer, miRNA panels derived from liquid biopsies can differentiate benign from malignant lesions (131), while in prostate cancer, lncRNAs such as PCA3 have been established as clinical diagnostic markers (132). Furthermore, circRNAs exhibit significant potential as diagnostic and prognostic markers for central nervous system tumors, a property attributed to their high stability and tissue specificity (133). Similarly, the expression profiles of exosomal ncRNAs in hematological malignancies facilitate disease classification and monitoring of therapeutic efficacy, underscoring their broad potential in precision medicine (134). In the realm of treatment, strategies such as engineering exosomes derived from MSCs to be loaded with let-7c or targeting and inhibiting miR-222-3p have significantly reversed drug resistance in preclinical models (80). Furthermore, significant progress has been made in applying exosomal ncRNAs in adjuvant immunotherapy. As previously discussed, exosomal ncRNAs can modulate immune cells within the tumor microenvironment to foster an immunosuppressive state. Targeting these specific ncRNAs presents a promising strategy to reverse immune escape and enhance the efficacy of checkpoint blockade therapy (135). Additionally, certain exosomal miRNAs, including miR-192 and miR-21, have been shown to regulate post-vaccination immune responses, suggesting their potential utility as vaccine adjuvants or predictive biomarkers for vaccine efficacy (136). Despite these promising findings, the clinical translation of exosomal ncRNAs faces several challenges. Significant inter-patient heterogeneity presents a primary obstacle; for instance, in hepatocellular carcinoma, individual variations in exosomal miRNA expression profiles compromise their consistency as universal biomarkers (137). A second major challenge is the technical difficulty in detecting these ncRNAs due to their exceptionally low abundance in bodily fluids, which often leads to insufficient analytical sensitivity (138). The lack of standardized protocols for exosome isolation represents another prominent issue, as inconsistencies across different platforms hinder the comparability of research findings and clinical results. This is particularly problematic for central nervous system tumors, where the blood-brain barrier limits the concentration of exosomal nucleic acids in the periphery, and variability in detection sensitivity among methods further obstructs clinical application (139). Finally, the clinical utility of exosomal biomarkers requires validation in large-scale, prospective cohorts. When considering exosomes as drug delivery systems, additional complexities arise, including the need to standardize isolation, purification, targeting efficiency, and large-scale production, all of which complicate the regulatory approval pathway (140, 141). In the future, it will be essential to establish large-scale, multi-center cohorts to validate the diagnostic value of exosomal ncRNAs. Additionally, developing more sensitive and specific detection technologies (such as nano-flow cytometry) and enriching tumor-specific exosomes in conjunction with surface markers (such as prostate-specific membrane antigen, PSMA) will enhance the sensitivity and specificity of detection. Simultaneously, spatial transcriptomics or CRISPR-labeling tracking technologies can be employed to analyze the dynamic changes of exosomal ncRNAs during tumor progression, dormancy, and recurrence. This will contribute to uncovering the spatiotemporal patterns of their regulatory networks. Second, treatment strategies targeting exosomes must strike a balance between efficiency and safety.

From the perspective of mechanism research, the regulatory network of exosomal ncRNAs extends far beyond the solitary ceRNA model. In the future, it will be imperative to integrate multi-omics data (such as the circRNA-miRNA-mRNA co-expression network) to analyze their synergistic or antagonistic effects and explore their interactions with epigenetic modifications. For instance, exosomal lncA1BG-AS1 influences m6A modification by regulating ZC3H13, indicating that epigenetic regulation could be a crucial pathway for exosomal ncRNAs to exert their functions. Moreover, the role of exosomes in tumor cell quiescence remains ambiguous. Quiescent cells in the bone metastasis microenvironment sustain a quiescent state by transferring molecules such as miR-34a via exosomes. Targeting these “quiescent signals” could potentially prevent metastasis recurrence, offering a direction for the development of novel maintenance therapies. Furthermore, regarding the clinical challenge of bone metastasis in PCa, the “vicious cycle” mediated by exosomal ncRNAs offers multiple targets for intervention. On the one hand, utilizing exosomes derived from BMSCs to deliver anti-resorptive drugs or ncRNA antagonists (such as miR-375) can directly disrupt the osteogenesis-tumor interaction. On the other hand, modulating exosomal circ-DHPS or lncAY927529 could reverse the pro-metastatic microenvironment. Additionally, inhibitors of exosomal microRNA (miR-92a-1-5p) that target osteoclast activation could potentially restore bone homeostasis and enhance the quality of life of patients. These certainly offer potential directions, yet there remains a significant distance to traverse before these goals can be truly accomplished.

In summary, exosomal ncRNAs serving as the “molecular bridge” within the TME of PCa, drive disease progression by facilitating complex intercellular communications and offer unparalleled opportunities for accurate diagnosis and targeted treatment. Despite the challenges they still encounter in exosome isolation techniques, heterogeneity analysis, and clinical translation, through interdisciplinary collaboration and technological innovation, exosomal ncRNAs are hold potential to be a key contributor to overcoming the treatment predicament of PCa. Future research endeavors should concentrate on unveiling their dynamic regulatory networks, formulating effective targeted strategies, and facilitating the implementation of personalized treatment regimens. By decoding the molecular language of exosomal ncRNAs, we can refine diagnostic precision, guide individualized therapies, and ultimately improve survival and quality of life for patients with advanced PCa.
